# The Challenges and Future Perspective of Woven Kenaf Reinforcement in Thermoset Polymer Composites in Malaysia: A Review

**DOI:** 10.3390/polym13091390

**Published:** 2021-04-25

**Authors:** Ching Hao Lee, Abdan Khalina, N. Mohd Nurazzi, Abdullah Norli, M. M. Harussani, S. Ayu Rafiqah, H. A. Aisyah, Natasha Ramli

**Affiliations:** 1 Institute of Tropical Forestry and Tropical Products, Universiti Putra Malaysia (UPM), UPM Serdang, Selangor 43400, Malaysia; ayu.rafiqah@yahoo.com (S.A.R.); a.humaira.aisyah@gmail.com (H.A.A.); iamtasha89@gmail.com (N.R.); 2 Centre for Defence Foundation Studies, Universiti Pertahanan Nasional Malaysia (UPNM), Kem Perdana Sungai Besi, Kuala Lumpur 57000, Malaysia; mohd.nurazzi@gmail.com (N.M.N.); norli.abdullah@upnm.edu.my (A.N.); 3 Advanced Engineering Materials and Composites (AEMC), Department of Mechanical and Manufacturing Engineering, Universiti Putra Malaysia (UPM), UPM Serdang, Selangor 43400, Malaysia; mmharussani17@gmail.com

**Keywords:** woven kenaf, thermoset polymer composite, Malaysia, processing methods

## Abstract

In this review, the challenges faced by woven kenaf thermoset polymer composites in Malaysia were addressed with respect to three major aspects: woven kenaf reinforcement quality, Malaysian citizen awareness of woven kenaf thermoset composite products, and government supports. Kenaf plantations were introduced in Malaysia in the last two decades, but have generally not produced much kenaf composite product that has been widely accepted by the public. However, woven kenaf fiber enhances the thermoset composites to a similar degree or better than other natural fibers, especially with respect to impact resistance. Woven kenaf composites have been applied in automotive structural studies in Malaysia, yet they are still far from commercialization. Hence, this review discusses the kenaf fiber woven in Malaysia, thermoset and bio-based thermoset polymers, thermoset composite processing methods and, most importantly, the challenges faced in Malaysia. This review sets guidelines, provides an overview, and shares knowledge as to the potential challenges currently faced by woven kenaf reinforcements in thermoset polymer composites, allowing researchers to shift their interests and plans for conducting future studies on woven kenaf thermoset polymer composites.

## 1. Introduction

Malaysia is a Southeast Asian country occupying parts of the Malay Peninsula and the island of Borneo. It is well known for its rainforests and non-seasonal weather. However, there is an overdevelopment of the urban area, which has been expanding its perimeter in recent decades. Hence, a huge region of rainforests is cut down for residential or commercial purposes every year. For this reason, the kenaf plantation project in Malaysia was initiated way back in 1999 by the National Economic Action Council (NEAC) as another industrial crop, and was intended to replace tobacco and prevent wood-logging for paper industry [1]. The kenaf plant was selected due to its fast growing, high productivity/land ratio, extraordinary photosynthesis rate and suitability for being planted in the Malaysian climate. Unfortunately, kenaf plantations in Malaysia face some difficulties, such as lack of skilled workers and low profit as a function of area of land [2]. Despite this, kenaf fibers provide superior enhancement to thermoset composites, especially in woven fabric form. The excellent impact resistance of woven kenaf has been used for applications in automotive structural components [3]. However, the slow development of this kenaf material has led to its failure as a pioneer in automotive green materials.

Thermosetting matrixes in composites are made up of two or more components, such as resin and hardener, where one of the components is a multifunctional monomer that crosslinks the material. High crosslinkages of cured thermoset polymer composites provide good performance, but cannot be reshaped after the curing process. Thermoset resins such as polyester, vinyl ester, and epoxy resins are very flexible, and are optimal choices with respect to providing products with desirable properties such as high modulus, strength, processibility at room temperature, and excellent thermal and chemical resistance. To combat and minimize plastic saturation issues, bio-based thermoset production is essential. Reinforcement with bio-based fillers such as natural fibers in a synthetic matrix will cause the product to exhibit certain degree of biodegradability. However, this is still not able to substitute the thermoset plastic products from a performance perspective. Global collaborations in the fields of research and advanced technology are seeking for bio-based thermoset resins to replace and substitute synthetic epoxies in advanced applications.

Thermoset composites are not only seeing an increased use in the applications ranging from racing cars and aircraft components to sporting goods, but they are also decreasing in terms of overall cost [4]. The fiber reinforcement gives thermoset composites excellent structural properties, while also making them inherently complex to manufacture. The composite manufacturing processes can be divided into two main processes: open and closed molding processing methods. Each of the processing methods has its own advantages and limitations. Therefore, the careful selection of the right processing method is as important as selecting the raw materials. In this review, we discuss the majority of thermoset composite production methods.

The uses of woven kenaf reinforcement are even compatible with synthetic fiber polymer composites. However, there are a few challenges that need to be addressed in order to widen the application of woven kenaf reinforced thermoset polymer composites in Malaysia. These challenges mainly come from three aspects: consistency of the woven kenaf reinforcement, the awareness of Malaysian citizens of woven kenaf thermoset composite products, and government support for knowledge delivery and research grants. It is understood that there will be minimal support from the government during and immediately following the COVID-19 pandemic. The underuse of woven kenaf thermoset composites may trigger some economic contraction in upstream industries, especially at kenaf plantations in Malaysia. Low demand for kenaf fibers will reduce profits, and this may cause farmers to change to other high-profit plantations. A lower supply of kenaf fibers will present difficulties for its use in mass produced products. Eventually, woven kenaf may not be able to be found in Malaysia. Green material development with kenaf would be terminated in Malaysia at the same time. Hence, this review sets guidelines for overviewing and sharing the knowledge, potentials and challenges currently faced by woven kenaf reinforcement in thermoset polymer composites, allowing researchers to shift their interests and plans for conducting future studies on the woven kenaf thermoset polymer composites.

## 2. Kenaf Plantation and Its Fibers in Malaysia

Kenaf (*Hibiscus cannabinus* L.) is a herbaceous plant that belongs to the family Malvaceae. In Malaysia, kenaf was introduced in the early 1970s, and was recognized as a possible renewable resource of fibrous fiber for industrial purposes in the late 1990s. Kenaf can be harvested within 4–5 months, and is a biodegradable and environmentally friendly crop, with one of the highest CO_2_ absorption rates. The height of kenaf is about 2.5–3.5 m, with a stem diameter of 25–51 mm [5]. Traditionally, kenaf fiber has been used for string, rope, cordage, padding material, sack and gunny. However, the utilization of kenaf fiber has been extensive and includes a variety of products such as mattresses, fuel, panels, and furniture parts, as well as pulp and paper.

**Figure 1:**
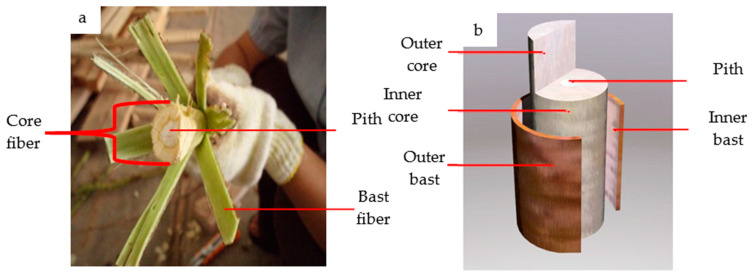
Kenaf outer and inner parts of the stem: (**a**) photograph, and (**b**) a sketch of the kenaf parts. Source: Figure reproduced with copyright permission from Juliana et al. (2012) [6].

Generally, the kenaf stem consists of an outer layer of bark and a core, which possess markedly different characteristics (Figure 1a,b). The bark is normally known as bast, and shows a fibrous and dense structure, while the core is woody and light, with a spongy structure in the middle area, which is known as pith. The ratio of bast to core is 30:70 of the stem dry weight. Kenaf bast and core are quite different with respect to their physical properties, anatomical structure, chemical composition, and mechanical properties. Table 1 presents a comparison of the most common fiber properties between these two parts. The variation in the values is due to differences in plantation location, retting process and species variation, which cause inconsistent kenaf fiber properties.

Kenaf can be used in the form of whole stem, bast or core. Due to the large differences in the properties of both fibers, fiber separation is required to ensure effective fiber processing, as well as excellent product manufacturing, since these fibers have different behaviors and react differently when exposed to heat, chemicals, water, etc. [9,15,16]. Decortication is the most common method used to separate fiber bundles into the long bast fiber and the core chip. On the other hand, retting is another preferred method, and involves a mechanism for removing the cell tissues and pectins that cover the bast fiber. The retting process can be performed using water, chemical, mechanical, or enzymatic retting processes [17]. The bast and core can then be processed into several forms, including particles, chips, chops, strands, short fibers, long fibers, yarn, woven fabric, and nano-sized particles.

Woven kenaf is one of the strongest reinforcement forms. The kenaf fiber strand is first twisted into a yarn form, and then woven into selected woven patterns. The woven textile is then cut into the desired shape for subsequent use in thermoset composite processing. The fabric counts and weaving patterns directly influence the end product’s performance [18]. However, there are no significant differences in tensile properties between different batches of woven kenaf fabrics [19]. Kenaf fiber provide superior impact strength compared to other natural fibers. Therefore, woven kenaf provides an effective enhancement of impact toughness that is comparable with woven Kevlar or other hybridization epoxy composites [20,21]. Structural components for automotive safety, such as passenger bumper beams, have been developed using glass and woven kenaf hybrid epoxy composites [3].

### Outstanding Impact Resistance of Kenaf Reinforcements

The idea of applying natural fibers in components in the automotive and other transportation fields was proposed with the aim of reducing the heavy weight of materials in order to minimize fuel consumption and carbon footprint. However, maintaining a similar strength has been found to be difficult using natural fiber polymer composites. Hybridization with synthetic fibers is one way to rapidly achieve the partial introduction of natural fibers in advanced applications. Among the various types of the natural fibers, kenaf fiber is one of the best reinforcements for improving impact resistance, and has been employed in the development of functional structural components in the automotive field. The partial use of woven kenaf have been found to provide comparable impact resistance to that of woven full glass composites, and previous studies have suggested its use in exterior components in the automotive field [22]. Hybrid composites with 10 layers of glass and kenaf fiber were able to absorb an impact energy of up to 40 J. Another high-velocity impact analysis showed that the kenaf hybrid composite was able to withstand an impact energy of up to 135 J, and thus presented anti-ballistic properties [23]. One previous study also commented that the insertion of kenaf fibers provided superior impact resistance and better energy absorbance in concrete [24]. Additionally, kenaf fiber treatments have been found to be highly effective with respect to impact resistance values. A three-fold improvement in impact strength was observed with alkali-silane treatment [25]. Both treatments improve the fiber/matrix interfacial bonding strength, allowing it to absorb more energy before breaking. Kenaf fiber is indeed a unique natural fiber available in Malaysia, and intensive research and development should be devoted to it.

## 3. Thermoset Resin

**Figure 2:**
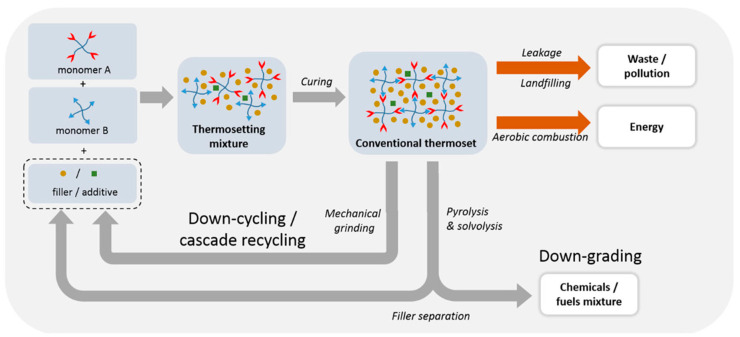
Schematic overview of conventional thermoset composite waste processing and recycling routes [33].

The thermosetting matrix in a composite is made up from two or more components, such as resin and hardener, where one of those components is a multifunctional monomer that crosslinks the material. It begins in a soft solid or viscous state that changes irreversibly into an infusible, insoluble polymer network upon curing to form a high crosslinking density [26]. As a result, thermosetting resins have very high impact resistance, but they cannot be reshaped after curing or polymerization [27]. However, cured thermosetting resins can be recycled effectively if ground into fine particles, which can then be used as fillers in new laminates or other products [28,29]. Figure 2 shows the recycling process for thermosetting plastics. Thermoset resins such as polyester, vinyl ester, and epoxy resins are very flexible, and are optimum choices for providing products with desirable properties such as high modulus, strength, processibility at room temperature, and excellent thermal and chemical resistance [30,31]. Table 2 shows some popular thermoset resins and their properties. Unfortunately, the combination of natural fibers and thermoset matrix results in weak interfacial bonding due to the presence of a hydroxyl group, limiting their optimal use in industrial application. Thus, a wide range of strategies have been applied to eliminate these deficiencies while providing interfacial bonding compatibility and strength between natural fibers and polymer matrix [32].

### 3.1. Epoxy Resin

Epoxy resin is the most important popular thermoset matrix used in highly developed composites. It is used in various fields, including adhesives, coatings, and structural materials [35]. Epoxy resings were initially discovered by Prileschajew in 1909 [36,37]. The term ‘epoxy’ refers to the simplest structure, which comprises a three-member ring, consisting of an oxygen atom bonded with two carbon atoms. They allow quick curing at wide range of temperatures from 5 to 150 °C. Despite their superior mechanical properties, low shrinkage and minimum internal stresses, which result from their high-intensity crosslinking, they are brittle in nature [38]. Epoxy resins are formed from a long molecular chain with epoxy functional groups at both ends. This is similar to vinyl esters, except that the absence of ester functional groups provide the epoxy with good water resistance [39].

**Figure 3:**
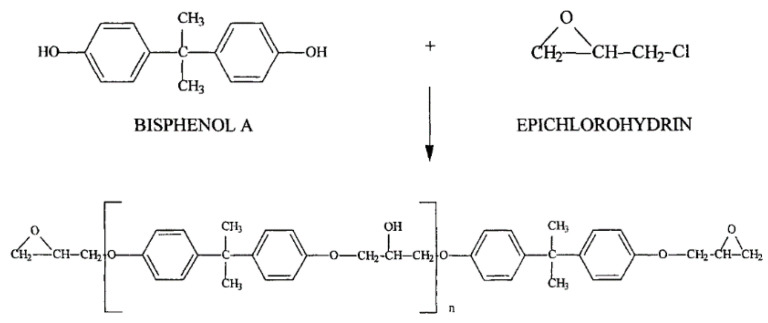
Typical Bisphenol A (BPA) epoxy resin [42].

There are three major types of epoxy resins: cycloaliphatic epoxies, epoxidized oils epoxies and glycidated epoxies. By far the most commercially available epoxy resins are those obtained by glycidation of bisphenol A (BPA) with epichlorohydrin, shown in Figure 3. The BPA rigid aromatic structure provides high performance to the cured resin or its composites [40]. Unfortunately, it may be the cause of several negative impacts on human health [41].

Epoxy resins need to be cured by a hardener rather than a catalyst. The hardener may function by means of either anionic or cationic homopolymerization, or be performed with a wide range of co-reactants including polyfunctional amines, acids, anhydrides, phenols, alcohols, and thiols [43]. Epoxy formulations also contain some additives and fillers. The aim of blending is to achieve the desired processing or final properties, or just to reduce cost [44]. The high crosslinking intensity may lead to a number of superior features such as high glass transition temperature, specific structure, high strength, good creep resistance, good dimensional stability and solvent resistance at high temperature. These properties can be further enhanced, and additional properties such as flame retardancy or electrical conductivity can be effectively introduced through the assimilation of co-monomers or additives [45,46,47].

Epoxy resins are widely used across a wide range of fields, from composite matrixes, general-purpose adhesives, and high-performance coatings, through to encapsulating materials [48]. In some cases, catalysts are required to increase the curing rate of epoxy resin. The catalysts are usually selected from tertiary amines or Lewis acids, and only a small amount is required. They initiate the ionic polymerization of the epoxy resin. The typical epoxy resin catalyst is boron trifluoride complexed with ethylamine. Such curing reactions result in the formation of tridimensional networks with a broad spectrum of performances, depending on the nature of the curing agent, and the extent and density of crosslinking [49]. Additionally, the heat level applied to the epoxy mixed with the hardener as a curing accelerator is related to the strengthening of the material through the crosslinking of polymer chains, which can be achieved by conventional heating, electron beams, chemical additives or accelerated curing (e.g., microwave, radiofrequency, or ultra-violet radiation) [50].

Furthermore, higher storage modulus and better thermal stability have been detected with the addition of natural fibers into epoxy composites, where they were a crucial factor in the composite’s elasticity [51]. Additionally, natural fiber reinforcements show superior mechanical properties, as well as a good strong interface between fiber and matrix [52,53]. Hydrophilic natural fibers have been found to exhibit lower moisture absorption as a result of coating with epoxy resin [54]. Therefore, natural fiber-reinforced epoxy composites may produce good properties in terms of mechanical and thermal characteristics.

### 3.2. Unsaturated Polyester Resins (UPR)

Unsaturated polyester resin (UPR) is one of the most exciting and common resins in the composites industry. UPRs are a type of high-performance engineering polymer that can be used in a variety of applications, including as a matrix for glass fiber composites [55]. UPR can be classified into five groups according to its structure, and vinyl ester resin is the most well-known group for composite matrixes [56].

UPR possesses a linear polymer structure with an ester bond and an unsaturated double bond, from the polycondensation of aromatic dicarboxylic acid. The viscosity can be reduced by applying reactive diluent, usually styrene at a temperature of 190–220 °C [49]. Styrene monomers and the UPR double bonds form free radical copolymerization to construct a rigid three-dimensional crosslinked structure [57,58,59]. An inhibitor is added to the resin to provide fast curing and long storage life, and to mitigate catalyzed or uncatalyzed drift, unwanted colors, odors and/or side effects. In comparison to epoxy, UPR lacks a hydroxyl group in its backbone chain, which results in poor bonding with natural fibers, causing significant shrinkage upon curing. Nevertheless, it still widely used for natural fiber composites [60].

There have been studies directed towards the development of composites using plant fibers reinforced with UPR, showing low strength and tending to absorb more water due to the incompatibility between the hydrophilic natural fibers and the hydrophobic UP. Good interfacial adhesion is essential to achieve superior mechanical properties and good water resistance [61]. The evaporation of residual water tends to create bubbles and voids within the composite and weaken the interfacial adhesion, thereby reducing the strength of the composite. In addition, heat treatment may also affect the morphology and composition of the fibers to some extent [62]. The composite is made through the combination of pretreated kenaf bast fibers with UPR as a matrix using a resin transfer molding process, resulting in a woven kenaf mat. Results have shown that this treatment was able to remove some impurities and amorphous content on the fiber surface, thereby increasing the tensile strength and modulus of the composite [63]. Yanjun et al. (2010) studied the reaction of UPR when vinyl silane was added to composites. The results showed an increased Young modulus, but the other mechanical properties did not change [62]. It’s possible that the increase in those properties can be attributed to a decrease in the hydrophilicity of the fibers after the fiber treatments. There was also improved interfacial bonding between the fiber and the matrix [64]. Supranee et al. (2008) developed a composite from treated sisal fiber and UP resin. The fiber treatment showed increases in the tensile, flexural, hardness and impact strength of the composite due to improved interfacial adhesion between the UPR and the fibers [65].

### 3.3. Vinyl Ester (VE) Resins

The addition reaction of epoxide resins with α-β unsaturated carboxylic acids produces a high-performance unsaturated resin, VE resin, and it is grouped among the unsaturated polyester resins [66]. Solvent storage tanks, sewage pipes, architecture and renovation, coatings, structural components in vehicles, swimming pools, and marine composites are all made using VE polymers. VE has the greatest flexibility due to its easy recycling processes. Hence, its products can be recycled several times over the course of their useful lives by turning them into post-industrial recycled products.

Rodriguez et al. (2007) examined the influence of 5 wt.% NaOH treatment at room temperature for 24 h on the mechanical properties of 30 vol.% woven jute/epoxy/VE composites fabricated using the vacuum infusion technique. Good properties were achieved for the surface-treated fiber insertion with the exception of in the impact analysis [67]. Similar findings have also been reported on silane-treated sisal-reinforced fabric in VE composites [68]. Additionally, it has been demonstrated in kenaf fiber-reinforced vinyl ester composites that increasing the amount of filler loading increases the tensile and flexural properties. These superior properties were mainly due to the excellent adhesion of fibers on the VE matrix [69].

### 3.4. Bio-Based Epoxy and Other Thermoset Resins

To counteract and minimize plastic saturation issues, bio-based thermoset production is essential. Reinforcement with bio-based fillers such as natural fibers will result in the synthetic matrix exhibiting a certain degree of biodegradability. However, there is a global demand for fully bio-based products in order to leverage the damaging activities carried out in past decades. A bio-based thermoplastic polymer, polylactic acid (PLA), has successfully demonstrated the possibilities of commercialized bioresins. However, it is still not a feasible substitute for thermoset plastic products from a performance perspective. As a breakthrough in epoxy resins, bioepoxies were developed to solve the general shortcoming of synthetic polymers: that they are non-biodegradable.

Bioepoxies are epoxidized from natural oils, where the presence of fatty acids in a triglyceride structure polymerizes into crosslinked biopolymers [70,71]. Thermal or cationic copolymerization of these plant oils with styrene or divinylbenzene is usually carried out to obtain thermosetting bioresins, ranging from elastomers to rigid products, depending on the stoichiometry, type of plant oils, and/or co-monomers. The application of bioepoxy has been reported to provide higher mechanical and thermal characteristics, although the opposite outcomes have also been obtained [72,73]. Nevertheless, bioepoxy composites were recorded as having good biodegradability properties [74,75].

At the same time, other bio-based thermoset resins have undergone intensive development. A series of prepolymers was synthesized using various saturated diacids to produce bio-based UP resins [76]. Itaconic acids containing carbon-carbon double bonds and two carboxyl groups have been demonstrated to have the capability to produce bio-based UP resin [77]. Similar thermomechanical properties were presented when compared to commercial UP resins [78]. Additionally, bio-based VE resins made from plant oils and carbohydrate biomass building blocks have been reviewed previously [79]. Lignin-based VE show great prospects for the development of bio-based VE resin because of their chemical structure, which is similar to that of styrene [80]. Overall, comparable thermal stability and thermo-mechanical properties have been reported [81].

The insertion of bio-based thermoset polymers into polymer composites is still not popular, due to the deterioration of characteristics in almost all respects. However, there is global collaboration in research and advanced technology aids with aim of replacing and substituting synthetic epoxies with bio-based thermoset resins in advanced applications.

## 4. Thermosetting Polymer Composite Processing

Advanced composite materials are popular in structural/functional product applications due to their superior strength [82,83]. As a result, composites are employed in products such as vehicles, aircraft parts, and sports goods, resulting in lower overall costs and weight. With this high demand for composite products, the production process becomes a crucial factor. Fiber-reinforced thermoset composites have excellent structural properties, but they are also difficult to produce [84]. Open and closed molding are the two primary types of composite manufacturing techniques. Open molding processes are those in which the resin is exposed to the environment during curing, while in closed molding processes, the opposite is the case.

**Figure 4:**
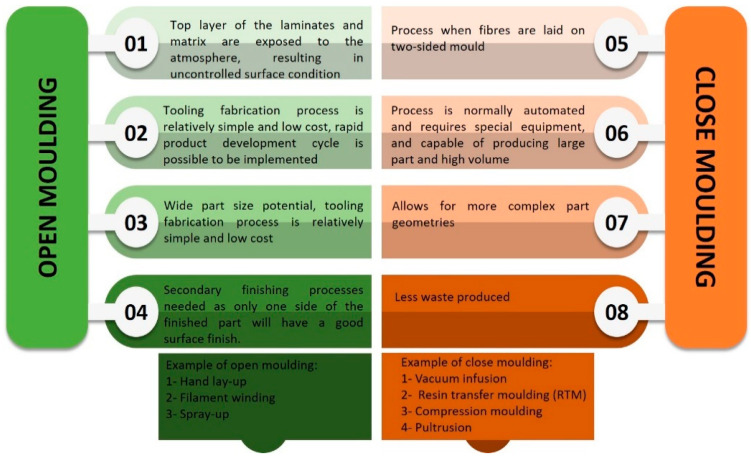
Comparison between open molding and closed molding.

The open molding method is typically used for a wide variety of materials that cannot be manufactured using automated processes, or for low-volume parts where the higher mold costs of automated processes cannot be justified [85,86]. Closed molding, on the other hand, is used to manufacture large quantities of precision parts. It is normally automatic, which necessitates the use of specialized machines. Dry reinforcements are laid in the base mold in closed molding, and resin is poured into the closed cavity with the assistance of a pressure pump or vacuum. Closed molding methods have more benefits, including lower material and disposal costs, a more consistent, repeatable procedure, and shorter cycle times, all of which contributes to improved efficiency while reducing labor costs. Since the resin is cured in a closed molding device, pollutants and waste are minimized [87]. Figure 4 depicts a comparison of open and closed molding, as well as processing instances. Each procedure will be discussed in depth.

### 4.1. Open Mold

#### 4.1.1. Hand Lay-Up Process

The hand lay-up process is the most common and widely used, especially for lab-scale sample preparation. It involves manually laying down individual layers or plies in a form of reinforcement known as prepreg. The reinforcement is pre-impregnated with resin and bundled into tows and arranged either in a single unidirectional ply or woven together. After that, a roller is used to expel trapped air bubbles and force thermoset resin into the reinforcement layer in order to enhance the dispersion of resin and the wettability of the reinforcement layer [88,89,90]. The high-quality complex features and the relatively low start-up cost of this method make it highly adaptable to new parts and design changes. However, the low production rate and the high dependence on worker skill limit the popularity of the hand lay-up process. There is a high probability that discrepancies between parts may result from human variation [91,92]. Nevertheless, superior mechanical properties are still achievable in hand lay-up specimens, and it is preferrable in the replacement of automotive parts [93].

The mechanical properties of composites fabricated using the hand lay-up method are directly influenced by the stacking sequence, fiber volume fraction and morphology, as well as the curing process. The process is capable of producing complex high-performance parts, but it can be an expensive and variable process. This is because the manufacturing process is highly dependent on worker skill. Elkington and colleagues described the hand lay-up process in detail, and identified a set of techniques that form the basis of this process [94]. The techniques used for preparing the prepreg woven fibers were found to be highly important. Shearing the reinforcement weave tended to have stronger links to specific tasks, but was also dependent on drape pattern, shear distribution and mold geometry. It was also noted that more techniques may be required for changing mold inclination and shear angle. However, this technique is still currently applied in large parts of the composite manufacturing industry, including in boat, bike chassis and sport goods production [95]. Manufacturers are always seeking other suitable methods that incorporate automation in order to reduce variations in cost and quality.

#### 4.1.2. Filament Winding Process

The filament winding technique is one of the oldest manufacturing techniques for composite materials. It is a process for fabricating composite structures in which continuous fiber reinforcements (filament, wire, yarn, tape, or other), either previously impregnated with thermoset resin or impregnated during filament winding, are placed over a rotating form or mandrel in a prescribed way to meet certain stress conditions. Fire extinguishers and cooking gas and oxygen tanks are examples of products made using filament winding [96]. Thermoset resins such as epoxy and unsaturated polyester are generally used as binders for reinforcements. The reinforcement can be applied to the dry roving at the time of winding, which is known as wet winding. Wet winding is the most widely used and cheapest method among the three types of filament winding (dry, wet and semi-dry filament winding). The fibers may also be applied in tow or tape prepreg forms. The wound product is usually cured at elevated temperatures without the use of vacuum bagging or autoclave compaction, then the procedure is finished by cutting off the trimming, mandrel and/or other finishing operations.

Untwisted fibers are often used in the aerospace industry, while twisted fibers are added by the manufacturer of the composite when the untensioned fiber is introduced. The manufacturing of pipes and gas tanks is also commonly performed using this process. The mandrel can be cylindrical, spherical, or any other shape, as long as it does not have a re-entrant (concave) curvature, although several manufacturers have been able to incorporate complex re-entrant curves in filament-wound structures [97]. In this process, there are three types of winding pattern: hoop, helical, and polar [27].

The challenge of reducing cycle time has become a major task in filament winding innovation over the past few years. Single tow fiber winding was developed into the multi-filament winding technique (MFW). The application of multi-axial robotic arm winding also improves the flexibility of the process, allowing products with more complex geometries [98]. Multi-angle filament winding has also been demonstrated to offer a better strength profile [99]. Certain winding orientations have been reported to provide improved energy absorption [100,101].

However, there are normally voids present in filament winding products, which significantly reduces their strength. A new tow impregnation system presented by Lasn and Mulelid successfully reduced the void content [102]. It was also confirmed that the composite microstructure was relatively insensitive to the winding speed and to the final cylinder thickness.

#### 4.1.3. Spray Up Process

Spray up is a process where compressed air is supplied through a spray gun, spraying the chopped fiber and resins in an open mold [103]. Sometimes a roller is applied on the sprayed surface to remove trapped air between the layers. Repeated spraying processes may be applied to achieve the desired part thickness. The cured part is detached from the mold for further manufacturing processing. Due to the spray nature of this process, products with complex geometries can be produced in an effective way. The final quality of the composite product is highly dependent on worker skill and competence. A skilled worker is able to generate a wide and stable angle variation with smooth performance [104], producing the desired thickness with a low coefficient of variation compared to non-expert workers. Hence, this method is more suitable for lower load-carrying components such as boats and trucks fairing.

Recently, the spray up process has been integrated with automated robotic arms to produce random discontinuous fiber composites (DFC) [105]. The linear robot speed has a major effect on the mass flow rate of the resin mode, but only a minor effect on the fiber mode [106]. A speed of 0.75 m/s and a 70° spray angle were identified as the most optimum parameters for achieving positive enhancement with the lowest coefficient of variance. At the same time, hybridization increased the composite tensile strength by 171%, reducing the density and increasing the thermal properties [105].

### 4.2. Close Mould

#### 4.2.1. Vacuum Infusion Process

Vacuum infusion, also known as vacuum-assisted resin transfer molding (VARTM), is a technique that uses vacuum pressure to push resin into laminate. Reinforcements are placed on a vacuum bag-covered mold, and flow media is applied to ensure a smooth and even flow of resin. If a full vacuum is obtained, usually at 25 mmHg, resin can penetrate the laminate through a special inlet tube [86]. The specific applied vacuum causes the resin to disseminate well and to disperse and be sucked through the perforated tubes (outlet tube) over the fibers in order to consolidate the laminate structure. This process leaves no space for the entrapped bubbles in the composite structure, making it popular for manufacturing large objects like boat hulls and wind turbine blades [107,108].

There are many differences in results between hand lay-up and vacuum infusion [109]. The vacuum infusion process provides higher flexural strength and interlaminar strain energy release rate compared to the hand lay-up process, making it a promising fabrication process [110]. Fracture surface analysis (SEM) shows that vacuum-infused composite samples exhibit a void-free matrix phase, resulting in high tensile properties. It has also been reported that the distribution of fibers is better than that in resin casting processed composites [111].

Hazardous styrene evaporates easily from the resin used during the hand lay-up laminating process, but this can be avoided when using vacuum infusion. Additionally, the vacuum assists in eliminating air bubbles and presses the lamination to the thinnest possible thickness by means of partial pressure, producing a high fiber-to-resin ratio. This results in a product with high strength-to-weight ratio. It also offers an unlimited preparation time before the thermoset resin starts to cure [109]. Additionally, the preparation in the dry state (without the resin) offer cleanliness to workers.

#### 4.2.2. Resin Transfer Molding (RTM) Process

RTM is a closed molding process in which the reinforcement material is laid in a closed mold and resin is pumped under pressure. This process allows complex parts with good surface finishes on all dimensions. The dry mold preparation, similar to the hand layup process, enables the flexible configuration of materials and orientations. The mold’s cavity volume is filled up with resin to produce the desired component thickness. The advantages include high fiber volume, low void contents and controlled surface finishing on both sides of the panel. In addition, the processing parameters of RTM can be altered, including pressure and temperature, before, during and/or after resin injection, to control the laminate’s quality. Hence, the product is able to obtain good surface finish, low void content, good dimensional tolerances, nominal tooling cost and a good range of available resin systems.

RTM is mainly used for the production of high-strength composite materials. In this process, resins and additives are kept separately and only mixed before processing (by adjusting the percentage of additives mixed to the resin). The mixture is then poured into the mold cavity, where the fiber reinforcements are placed into position. The increased mixture viscosity and poor dispersion of fillers make good fabrication difficult in practice [112]. Hence, a vacuum is created throughout the mold cavity by using a vacuum pump at the vent position. The negative pressure generated drives the even spread of resin throughout the mold cavity, thereby producing strong composite products. The RTM process produces products with promising quality, but 24 h of curing at room temperature, followed by a post-cure at 80 °C, is highly recommended [113]. Ultrasonic attenuation has been applied to reduce porosity for better fiber wetting [114]. Additionally, the emission of volatile components in the resin is low, because of the closed mold process [115].

**Figure 5:**
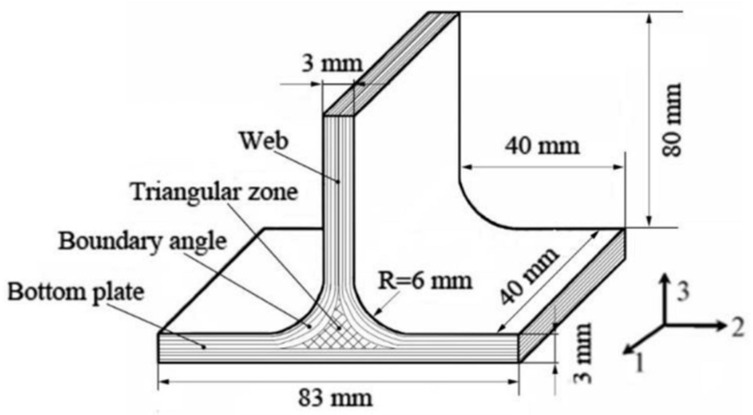
Composite joints with a triangular zone and boundary angle are producible using the RTM process [117].

Joint conditions are always a nightmare for resin transfer molding (RTM), which is a mass production process that can replace autoclave processes, and composite lap joints are extensively used in composite structures. Composite joints with fillers (Figure 5) are used to strengthen the joints, and these can be produced using the RTM process. The triangular zone filler and the boundary angle radius have been found to have a significant influence on the joint’s mechanical properties, reducing the shear and peel stresses generated. On the other hand, the fiber stitching process has been found to be effective in improving and complementing the strength of RTM-produced products. Its stitching intervals and patterns have been found to be important factors [116].

#### 4.2.3. Compression Molding Process

The compression molding process is a composite manufacturing process normally used to produce composite components in high production volumes, such as those required for automotive components. The compression molding process also allows the use of long fiber reinforcements in complex-shaped composite structures. The compression molding process enables flexibility of part design, as well as features such as inserts, ribs, bosses and attachments. The unidirectional reinforcement produces superior strength in one specific direction.

There are two types of compression molding process: cold compression and hot compression molding. Hot compression molding is utilized for thermoplastic polymer composite production, where heat is needed to melt the polymer. However, for some thermoset composites, heat is applied to accelerate the curing process and enhance the mechanical properties [118]. However, one of the major difficulties in processing fast-cure thermoset resins is their strong exothermic reaction during curing. Overshooting the temperature and large temperature gradients may cause shrinkage and residual stresses. To solve this matter, several models have been developed, showing the importance of the resin injection strategy [119]. For cold molding, the resins are maintained, pressed in cold molds until cured. The longer pressing period reduces the porosity of composites, but may be inappropriate for mass production [120]. Additionally, bonding of the material to the mold halves (mold sticking) is another issue that cannot be ignored. Insufficient mold temperature or cure time, mold wear, high amounts of charge weight, or inappropriate mold surface may cause the composite to stick to the mold [120].

#### 4.2.4. Pultrusion Process

**Figure 6:**
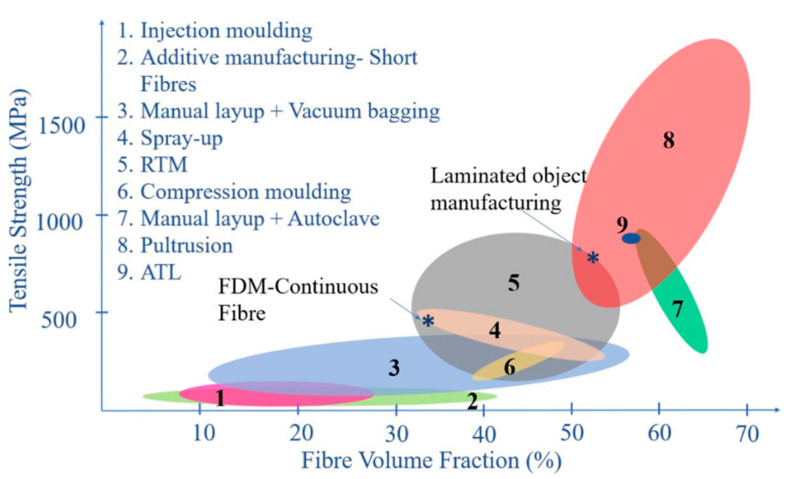
Tensile strength of various short fiber-reinforced polymer composites with different fiber volume fractions. (RTM: Resin Transfer Molding; ATL: Automated tape laying; FDM: Fused deposition modelling) [124].

Pultrusion is one a well-known polymer composite manufacturing technology that is based on pulling-extrusion techniques. It allows continuous production of a constant cross-section profile by using long fiber reinforcements. The fibers can be impregnated with a liquid resin in resin bath or injection box before being rolled onto the mold, producing good fiber wetting. Consequently, it generates high-strength products, as seen in Figure 6. Nevertheless, pulling speed and die wall temperature profile are variables that influence the quality of pultruded products [121]. This is not limited to other factors like die length, fiber filling, fiber form and the kinetic properties of the resin [122].

High productivity and low operating costs are the main advantages of this processing method. However, the curing rate of the resin during the process must be carefully controlled in order to provide a high-quality product at the end of the process. Additionally, the viscosity of the resin must be taken into consideration in order to optimize the wettability and fiber matrix adhesion. Due to the exothermic character of the curing reaction, inside the composite, the increase in temperature can cause the degradation of the product [123]. A study by Fairuz et al. (2016) on the effect of filler loading on the mechanical properties of pultruded kenaf fiber-reinforced vinyl ester composites showed that as the filler loadings were increased to a significant amount, the mechanical properties started to drop, which was attributed to the increase in viscosity of the matrix and, in turn, the increase in porosity and decrease in wettability of the composite [69].

## 5. Kenaf Fiber-Reinforced Thermoset Composites

**Figure 7:**
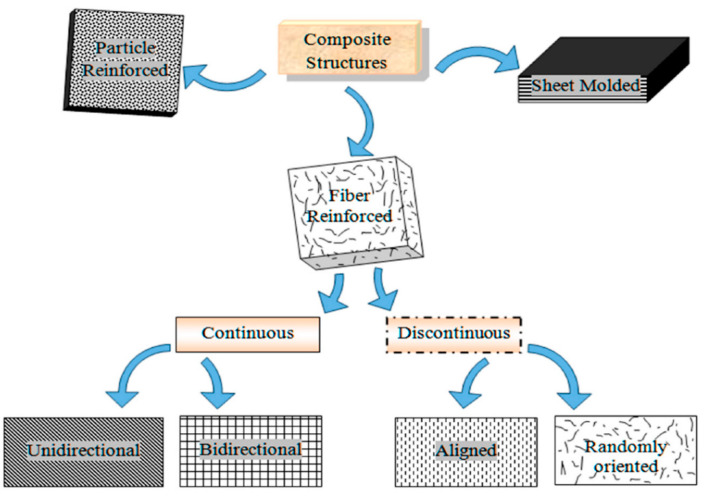
Composite classification based on reinforcement type. Source: Figure reproduced with copyright permission from Rajak et al. (2019) [125].

During the past several years, kenaf fibers have been shown to be suitable for composite production in many applications, including structural, non-structural, medical, furniture, automotive and flooring applications. Composite materials consist of two parts: (i) matrix, and (ii) reinforcement. Their classification is made in accordance with their content. Reinforcement is a filler, and is present in many forms. Based on their composition, composites can be divided into three major groups, as shown in Figure 7 [125,126]. Table 3 lists the utilization of kenaf fiber in various forms and their applications. Kenaf reinforcement in thermoset composites will be discussed in detail in the following section.

**Figure 8:**
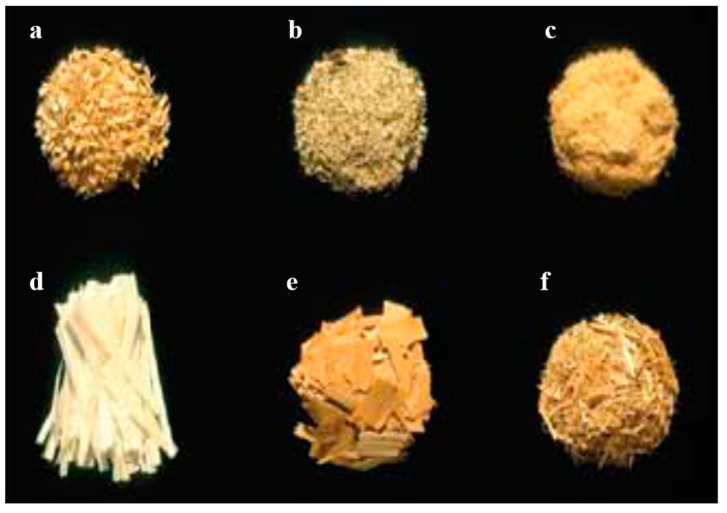
The types of form of kenaf fibers used in polymer composite production: (**a**) shavings, (**b**) sawdust, (**c**) fiber, (**d**) large particles, (**e**) wafers, and (**f**) strands. Source: Figure reproduced with copyright permission from Stark et al. (2010) [128].

In composite materials, short fibers are widely used for non-structural applications. Due to their ability to protect the crack matrix when used in composites, composites with short fibers as filler are expected to have superior quality [127]. Current research on the characteristics of short kenaf fibers is widespread. It is possible to produce kenaf composites in several sizes and types, as shown in Figure 8. One of the simple forms of kenaf is the kenaf chip, where the stem is directly processed in a chipper to produce kenaf chips. The chips can be a mixture of core and bast fiber, or the stem can be subjected to a decortication process prior to the chipping process. The chips are typically 30–50 mm in length, 25–40 in width and have a thickness of 4–6 mm. Element size and geometry largely determine the manufactured component and the efficiency of the product.

Kenaf-reinforced polymer composites have been used in a great deal of research work utilizing thermoset polymers. Studies have been carried out on a wide range of natural fibers as an alternative to wood fibers, and the results of studies have shown that fast-growing species such as kenaf can be harvested. During the last several years, kenaf in the form of short fibers has been shown to be suitable for polymer composite applications. Short bast fiber-based composites are frequently used for a variety of applications, such as in automotive applications and as construction materials. However, only a minority of thermoset composite studies have applied kenaf in short fiber forms. The main reason behind this is because the liquid nature of thermoset resins at room temperature make it difficult to achieve short fiber reinforcement. The longer curing time may cause the fibers and resin to segregate before the curing process is complete, when compared to thermoplastic polymer composites that possess a cold-formed structure. The inhomogeneous distribution of short fibers creates stress concentration spots, leading to earlier failure, before being subjected to the maximum load capacity. Nevertheless, Mohan and Kanny inserted short kenaf fibers in vacuum-infused epoxy composites. A compressive pressure of 8 bars was applied to the randomly dispersed fibers for one hour before the infusion of the epoxy resin, to ensure the flatness and uniformity of the composite.

Therefore, treatment is always required for kenaf fiber in order to remove non-cellulosic content from the kenaf fiber to achieve higher mechanical strength [147]. Additionally, before compression, cleaning the fiber surface using 5% of NaOH results in an improvement of both flexural and DMA properties as a result of the enhanced bonding ability and wettability of the fiber and matrix polymer. The effects of NaOH treatment on kenaf fiber have also studied using resin casting (RC) and vacuum-assisted resin infusion (VARIM) methods [111]. Increases in tensile, flexural and impact strength were reported resulting from the improvement in fiber-matrix adhesion and the fiber properties. By contrast, by utilizing the VARIM process, the tensile and flexural properties of the composite were improved when compared to the RC technique.

One of the favorable forms of natural fibers is continuous fiber, which refers to long fibers, which are lengthy compared to short fibers. The main reason for using long fibers is that continuous fibers are easy to orient and process, while also providing higher mechanical properties compared to short fibers with random orientation [3,148,149,150]. Their use results in an improvement in tensile modulus, due to the fiber arrangement along the load direction [150].

### Woven Kenaf Reinforced Woven Polymer Composites

Currently, there is a growing interest of the use of woven material from natural fibers in composite production due to its superior mechanical properties, flexibility and stability. This composite type is widely referred to as textile composites, and consist of technical textiles rather than traditional textiles. The woven fabrics are created from bundles of yarn that consist of numerous fibers by means of the weaving technique. Several studies have been conducted on the performance of woven kenaf fiber-reinforced polymer composites, finding that the linear density of the yarn and the structure of the weave have a major effect on the composite’s tensile properties [151,152].

On the other hand, reinforcement with woven kenaf has been demonstrated to be highly suitable for external medical equipment due to its superior enhancement of mechanical properties, especially with respect to impact resistance [153,154,155]. The woven kenaf reinforcement acted as a shield for resisting the spread of cracking [156].

Fabric parameters, such as fabric counts and weave designs, have been found to significantly influence the mechanical properties of woven kenaf hybrid-reinforced thermoset composites [157]. Plain woven kenaf with a loose structure had the highest flexural modulus strength characteristics, while tight fabric exhibited greater tensile and impact strength. At the same time, it was found that higher content of kenaf fiber material increased the thermal performance of the hybrid composites; the composite was more durable, with analysis demonstrating an elevated decomposition temperature [158]. Additionally, various experiments have been carried out on the ballistic properties and characteristics of woven kenaf fiber-reinforced polymer composites [159]. The addition of woven kenaf was found to lead to an increase in composite thickness and density, thus increasing energy absorption, as well as ballistic velocity [160]. Hence, it was proven woven kenaf improves impact energy absorption in composites, and multiple studies have employed woven kenaf in studies on ballistic-proof materials [142,161]. Woven kenaf has demonstrated superior reinforcement ability in previous studies. Therefore, the sustainability of its development should be maintained in Malaysia. However, there are numerous challenges faced by woven kenaf thermoset polymer composites in Malaysia.

## 6. Economic Value, Challenges and Future Perspective for Woven Kenaf Thermoset Polymer Composites in Malaysia

Kenaf fibers have a very high economic value, ranging from upstream to downstream industries. The global market for kenaf fibers is expected to reach US$854 million by 2025 [162]. It is an excellent raw material for papers, and hence has very high demand within this industry. Hence, the growth of the kenaf industry will ultimately lead to a boosting of the socioeconomic status of the Malaysian population, thereby reducing or eradicating mass poverty, deprivation, and underdevelopment in local communities. Unfortunately, there is a total of nine categories of issues and elements raised by farmers, low production output being the main concern [163]. The turnover rate for kenaf plantations varies from 5 to 58 years based on financial analysis [164]. To solve this issue, Malaysia’s former Primary Industries Minister urged the formation of a production chain ranging from kenaf crop cultivation up to industrial stakeholders [165].

**Figure 9:**
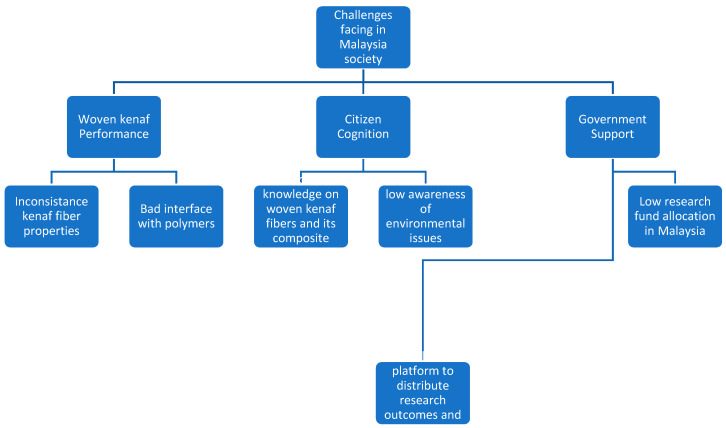
The challenges faced by woven kenaf thermoset polymer composites in Malaysia.

A complete supply-demand environment would be expected to produce higher price for kenaf fibers. Regrettably, woven kenaf thermoset polymer composites seem to have no value at the commercial stages. The use of woven kenaf reinforcement is even compatible with synthetic fiber polymer composites. However, there are several challenges that need to be addressed in order to broaden the application of woven kenaf reinforced thermoset polymer composites in Malaysia. Figure 9 summarizes the challenges faced by woven kenaf thermoset polymer composites in Malaysia.

Woven kenaf fiber is similar to other natural fibers, its properties are mainly governed by the chemical components in the fiber. However, high degrees of inconsistency were found in the chemical components among individual kenaf fibers, meaning that the properties vary from fiber to fiber. This has led manufacturers to step away from selecting woven kenaf fiber as a substitute for synthetic fibers, which are identical in all aspects. Other than this, the hydrophilic nature of woven kenaf was found to be incompatible with hydrophobic polymers. Even worse, though, was that insertion of woven kenaf fibers led to higher water absorption properties [166]. Moisture absorption accelerates the biodegradation process, resulting in earlier failure of the composite’s geometrical integrity and functionality. This is totally intolerable for application in advanced products. Sudden malfunction may cause the loss of huge amounts of money and/or valuable lives.

Even though the majority of reports on surface treatments describe a positive enhancement of the woven kenaf thermoset composite properties, there is an in the overall incurred cost and production cycle-time [167]. This has presented industry with a selection dilemma. Fortunately, awareness of environmental responsibility is improving, nowadays. The use of kenaf green fibers has become a gimmick or a selling point used by companies to improve their reputation. Nevertheless, industrial stakeholders should take the initiative and use the woven kenaf fiber reinforcement in Malaysia, as this is a future path of material development. Industrial collaboration and industrial funding are important criteria for the development of woven kenaf thermoset composite product, especially at the commercialization stage. Comments from industry collaborators are highly valuable, because they understand the needs of consumers with respect to the product.

Unfortunately, the awareness of Malaysian citizens of environmental issues is still insufficient. Malaysia generated 4.0 million tons of solid waste in 2019 [168]. The plastic disposal portion (especially single-use masks, gloves and other personal protective equipment (PPE)) were expected to be increased dramatically from 2020 onwards due to the COVID-19 pandemic. From a psychology perspective, people tend not to buy products that they don’t know about. Hence, if citizens have a greater knowledge of kenaf green woven fibers and the fact that woven kenaf thermoset composite products have high compatibility in advanced applications, it will reduce the saturated municipal solid waste situation in Malaysia. Furthermore, global researchers are currently developing PPE made of natural fibers in order to reduce the dependence on conventional plastic [169]. Malaysian researchers could treat this as an opportunity to study disposal masks made from the natural fibers of kenaf.

Now is the perfect moment to introduce woven kenaf thermoset composites to the public. This would require full support from the government and universities. The lack of suitable platforms for disseminating research achievements has meant that the innovations remain among the research society, and fail to be shared with the public. Government should create visible platforms for delivering Malaysian research breakthroughs to citizens of all levels. Social media, newspapers, public campaigns and/or community activities are some of the effective channels for embedding achievements by researchers related to woven kenaf composite [170]. On the other hand, Malaysia has committed a very low amount of research funding in the annual expenses budget compared to other countries [171]. Insufficient funding will slow down the progress of research works, limiting both recruitment and sharing at international conferences.

Unfortunately, research funding is currently difficult to obtain in Malaysia and around the world, during this COVID-19 pandemic period. It is understandable that governments have to focus on the recovery of the social economy [172]. As a future perspective, progress should be made on multiple fronts to introduce woven kenaf thermoset composites to the Malaysian population, and also at the international level. Therefore, this review overviews and shares the knowledge, potentials and challenges currently faced by woven kenaf reinforcement in thermoset polymer composites, allowing researchers to shift their interests and plans for conducting future studies on the woven kenaf thermoset polymer composites.

## 7. Conclusions

In conclusion, kenaf plantations are well established in Malaysia, yet they are facing some difficulties such as lack of skilled workers and low profit by area of land. In spite of this, kenaf fibers provide superior enhancement to thermoset composites, especially in woven fabric form. The excellent impact resistance of woven kenaf has been used in automotive structural components. Thermosetting matrixes in composites are made up of two or more components, such as resin and hardener, where one of the components is a multifunctional monomer that crosslinks the material. Cured thermoset polymer composites provide good performance, but cannot be reshaped after the curing process. Polyester, vinyl ester, and epoxy resins are optimal thermoset polymer choices for use in products with superior performance. However, conventional thermoset polymers are not biodegradable. To combat and minimize plastic saturation issues, bio-based thermoset products have been introduced. Composite manufacturing processes can be divided into two main types of process: open and closed molding processing methods. Each of these processing methods has its own advantages and limitations. Therefore, the careful selection of the right processing method is as important as selecting the raw materials.

Unfortunately, there are several challenges that need to be addressed in order to broaden the potential application of woven kenaf reinforced thermoset polymer composites in Malaysia, mainly stemming from: the consistency of woven kenaf reinforcement, the awareness of Malaysian citizens of woven kenaf thermoset composite products, and government support for knowledge delivery and research grants. It is understood that there will be minimal support from the government during and immediately following the COVID-19 pandemic. However, this review provides an overview and shares the knowledge, potential and challenges currently faced by woven kenaf reinforcement in thermoset polymer composites, allowing researchers to shift their interests and plans for conducting future studies on the woven kenaf thermoset polymer composites. In the future, it is hoped that woven kenaf thermoset polymer composites will be able to be applied as substitutes for some advanced materials that require high impact resistance, such as in the automotive and aerospace fields. At the same time, improved socioeconomic status of low-income families in Malaysia could be achieved as a result of there being a higher demand of woven kenaf.

## Figures and Tables

**Table 1:** Comparison of the some of the properties between kenaf bast and core. Sources: [7,8,9,10,11,12,13,14].

Properties	Unit	Bast Fiber	Core Fiber
Physical	Moisture Content (%)	73–75 (green stem)	
	Density (g/cm^3^)	1.20–1.50	0.10–0.20
Anatomical	Fiber Length (mm)	1.4–5.0	0.4–1.1
	Fiber Width (µm)	17.34	19.23
	Aspect Ratio	128	39
	Lumen Diameter (µm)	12–23	18–37
	Cell Wall Thickness (µm)	3.6	1.5
Chemical Composition	α-Cellulose (%)	55.0	49.0
	Holocellulose (%)	86.8	87.2
	Lignin (%)	14.7	19.2
	Ash Content (%)	5.4	1.9
	Extractives (%)	5.5	4.7
Mechanical	Tensile Strength (MPa)	295–1191	-
	Young’s Modulus (GPa)	22–60	-
	Elongation (%)	1.6	-

**Table 2:** Properties of thermosetting resin [34].

Type of Resin	Density (g/cm^3^)	Tensile Strength (MPa)	Tensile Modulus (MPa)
Phenolic	1.19–1.2	10	375
Unsaturated polyester	1.025–1.5	40–90	2000–4500
Vinyl ester	1.2–1.4	69–83	3100–3800
Epoxy	1.1–1.4	35–100	3000–6000

**Table 3:** List of reported studies on kenaf fiber reinforcement in various forms and its applications.

Kenaf Part and Form	Type of Thermoset Matrix	Processing Method	Applications/Products	References
Kenaf core chip, bast chip and industrial wood chip	Urea formaldehyde (UF)	Hand lay-up and hot pressing	Three-layer particleboard	[129,130]
Core particles	-	Hand forming and steam injection pressing	Binderless particleboard	[131]
Core particle	UF	Hand lay-up and hot pressing	Particleboard	[132]
Ground kenaf core	-	-	Filtration aid	[133]
Core particles	-	-	Animal bedding materials	[134]
Bast fiber	UF	Hand lay-up and hot pressing	Medium density fiberboard (MDF)	[135]
Core fiber	UF	Hand lay-up and hot pressing	Medium density fiberboard (MDF)	[136]
Kenaf stem fiber	UF	Hand lay-up and hot pressing	Medium density fiberboard (MDF)	[137]
Short kenaf fiber	Epoxy	Hand lay-up and compression molding	Composite component	[138]
Kenaf short fiber	Epoxy	Resin casting and vacuum-assisted resin infusion (VARIM)	Epoxy reinforced composite	[111]
Long bast fiber	Epoxy	Hand lay-up and hot pressing	Plastic composite for automobile structure	[139]
Kenaf and banana fiber	Epoxy	Hand lay-up	Composites for automotive manufacturing and packaging	[140]
Woven, unidirectional, non-woven of kenaf and Kevlar	Epoxy	Hand lay-up and compression pressure	Spall liners for military vehicles	[141]
Woven kenaf and Kevlar	Epoxy	Hand lay-up and compression pressure	Ballistic laminate composites	[142]
Kenaf fabric and aramid	Polyvinyl butyral	Compression molding	Combat helmet	[143]
Woven kenaf, woven flax, glass fiber, carbon fiber	Epoxy	Hand lay-up	Composite component	[144]
Plain woven kenaf and sisal fiber fabric	Epoxy	Compression molding	Composite for semi-structural materials in outdoor applications	[145]
Plain woven kenaf and sisal fiber fabric	Bio-epoxy resin	Compression molding	Composites for semi-structural applications	[146]
Kenaf yarn and glass fiber	Epoxy	Filament winding technique	Composite component for tube application	[100]

## Data Availability

No new data were created or analyzed in this study.
